# Milk Fat Globule Membrane Supplementation Promotes Neonatal Growth and Alleviates Inflammation in Low-Birth-Weight Mice Treated with Lipopolysaccharide

**DOI:** 10.1155/2019/4876078

**Published:** 2019-05-02

**Authors:** Shimeng Huang, Zhenhua Wu, Cong Liu, Dandan Han, Cuiping Feng, Shilan Wang, Junjun Wang

**Affiliations:** ^1^State Key Laboratory of Animal Nutrition, College of Animal Science and Technology, China Agricultural University, Beijing 100193, China; ^2^Department of Obstetrics and Gynecology, China-Japan Friendship Hospital, Beijing 100029, China

## Abstract

Impaired intestinal mucosal integrity and immunity are frequently observed in low-birth-weight (LBW) animals, which lead to inadequate growth and high neonatal mortality. However, the mechanisms of intestinal dysfunction in LBW animals are still unclear. Milk fat globule membrane (MFGM), a protein-lipid complex surrounding the fat globules in milk, has many healthful benefits for animals. Therefore, this study was conducted to explore the effect of MFGM supplementation on intestinal injury and inflammation in LBW mouse pups while being challenged with lipopolysaccharide (LPS). C57BL/6J LBW female neonatal mice were fed on breast milk and divided into four groups, including two normal diet groups (ND; CON group and LPS group) and the diet supplemented with two dosages of MFGM, namely, MFGM100 (ND plus MFGM at 100 mg/kg BW) and MFGM200 (ND plus MFGM at 200 mg/kg BW) from postnatal day (PND) 4 to PND 21. At PND21, pups from the LPS group, MFGM100 group, and MFGM200 group were injected intraperitoneally with LPS while the pups from the CON group were injected with equivalent volume of sterile saline. After 4 h of LPS administration, all pups were slaughtered and then the plasma, mid-ileum, and mid-colon tissue samples were collected. Our results showed that MFGM supplementation promoted the body weight from PND16 to PND21 and attenuated intestinal inflammation manifested by reduced histological damage, decreased secretion of TNF-*α*, IL-6, IFN-*γ*, and IL-1*β*, and improved oxidative stress characterized by increased SOD activity and decreased secretion of MDA. Expression of tight junction proteins (ZO-1, occludin, and claudin-1), MUC1, and MUC2 was increased in MFGM presupplemented groups compared to the LPS-challenged mice with normal diet. Meanwhile, the expression of proinflammatory cytokines and TLRs was decreased by MFGM presupplementation. Collectively, MFGM is a critical nutrient with an ability to improve the growth performance of LBW mouse pups, especially during the LPS challenge, by promoting the intestinal epithelial integrity and inhibiting inflammation through activating of TLR2 and TLR4 signals.

## 1. Introduction

Infants with low birth weight (LBW) have higher morbidity and mortality than normal birth weight infants during their neonatal period as an outcome of intrauterine growth restriction (IUGR) [1], which was defined as impaired growth and development of the mammalian embryo/fetus or its organs during pregnancy [2]. Despite advanced prenatal care for both mothers and fetuses, approximately 15% of human infants suffer from LBW worldwide [3] and approximately 15-20% of newborn piglets suffer from IUGR [4]. Neonates with LBW show impaired intestinal development, nutrient metabolism, and immune function in both human-beings [5, 6] and animal models [2, 7, 8]. Metabolic programming and the alterations in early physiological may proceed to intestinal dysfunction during the neonatal period [9]. During this critical window, nutrition is the principal contributor to the development of immune and metabolic and the establishment of microbes [10]. Epidemiological studies also indicated that the LBW humans or animals are in high risk of metabolic disease in their later life [11]. Therefore, exploring novel strategies to improve the development of LBW animals or human infants during their suckling period is paramount and has prospects for practical applications.

Milk fat globule membrane (MFGM), derived from the apical surface of mammary epithelial cells and composed of proteins and lipids [12], could protect the milk fat globules and retard their physical destabilization in milk [13, 14]. Several studies have found that supplementation of the MFGM could decrease the infection and inflammation in rodent models [15] and promote the gut mucosal integrity during lipopolysaccharide (LPS)-induced intestine inflammation in male BALB/c adult mice [16], suggesting that MFGM possess a function of antibacterial and anti-inflammatory activity [16]. In addition, MFGM has attracting attentions in related metabolic disorders in high fat diet induced obese mice [17]. MFGM treatment on human infants and neonatal piglets could improve the neurodevelopment and increase the cognitive as compared to the control formula, reaching a similar effect to those of breastfed infants [18–20]. However, previous studies showed no significant effects of MFGM supplementation on weight gain and feed intake of normal birth weight mice during early life and adulthood time [16, 21]. Whether MFGM can promote the growth of LBW newborns or alleviate the intestinal inflammation and injury induced by LPS challenge is unknown. We hypothesized that MFGM supplementation could attenuate the intestinal inflammation and damage in LBW pups, especially while being challenged with LPS. Therefore, the present study was conducted to investigate the protective effect of MFGM against intestinal injury in LBW mice during neonatal growth and LPS challenge, as well as the possible mechanisms.

## 2. Materials and Methods

### 2.1. Animals and Treatments

All experiments were performed in accordance with the Health Guide for Care and Use of Laboratory Animals by China Agricultural University. C57BL/6J female mice were obtained from Sibeifu Inc. (Beijing, China). All mice were housed under a 12 h light/dark cycle, a constant temperature (24°C), and 50% humidity. Pregnant mice were housed individually with* ad libitum* access to normal chow diet (KeAoXieLi Feed Co., Ltd., Beijing, China). At birth, pups were spontaneously delivered from female mice and their body weights were recorded. A low-birth-weight (LBW) pup was defined when its birth weight was lower than 2 SD (standard deviations) of the mean birth weight of their littermates [22, 23]. On postnatal day (PND) 4, LBW female mice (n = 32) were cross-fostered to adjust the litter size among these groups and were divided into four groups (n = 8 pups/group), including two normal diet groups (ND; CON group and LPS group) and the diet supplemented with two dosages of MFGM, namely, MFGM100 (ND plus MFGM at 100 mg/kg BW) and MFGM200 (ND plus MFGM at 200 mg/kg BW) from PND4 to PND 21. Meanwhile, body weight was measured every day and dosage of MFGM administration was adjusted. From PND4 to PND11, pups were administered in a volume averaged 20 *μ*L and 50 *μ*L from PND11 to PND21. At PND21, pups from the LPS group, MFGM100 group and MFGM200 group were injected intraperitoneally with LPS (10 mg/kg BW; E. coli serotype 055: B5, Sigma Chemical) while the pups from the CON group were injected with equivalent amount of sterile saline.

### 2.2. Tissue Sampling

After 4 h of LPS treatment, all the pups were anesthetized with tribromoethanol (500 mg/kg) and blood was taken to collect plasma and the mid-ileum and mid-colon were dissected. The blood was centrifuged for 15 minutes at 3000 rpm and plasma stored at -20°C. Intestinal tissues (mid-ileum and mid-colon) were harvested immediately and fixed in 4% buffered formalin overnight and processed for routine histological analysis. Meanwhile, the mid-ileum and mid-colon were washed with normal saline, snapped frozen in liquid nitrogen, and stored at -80°C prior to processing for ELISA and qRT-PCR analysis of the cytokines.

### 2.3. Histological Analysis

The paraformaldehyde-fixed mid-ileum and mid-colon were dehydrated in graded alcohol and embedded in paraffin wax. Then, hematoxylin and eosin (H&E) stained paraffin sections were viewed under bright field on a Zeiss Axio Imager microscope as outlined previously. Microscopic intestinal damage was observed in images using the measurement tool on CaseViewer software at 200x magnification. The degree of intestinal tissue damage was scored as described by Ji et al. [24] and Nishiyama et al. [25], where the extent of epithelial loss on intestinal villi and inflammatory infiltration was evaluated and included in the histopathological examination.

### 2.4. Plasma Inflammatory Profile Analysis

The concentrations of proinflammatory cytokines (TNF-*α*, IL-6, and IL-1*β*) were detected using enzyme-linked immunosorbent assay (ELISA) kits according to the protocol provided (eBioscience, CA, USA). Meanwhile, the concentrations of malonyldialdehyde (MDA) and superoxide dismutase (SOD) were analyzed by kits from Nanjing Jiancheng Bioengineering Institute (Nanjing, China).

### 2.5. RNA Extraction and Quantitative Real-Time PCR Analysis

Total RNA from mid-ileum and mid-colon were extracted using TRIzol kit (Invitrogen, Carlsbad, CA, USA) following the protocol. cDNA was obtained using PrimeScript™ RT Kit (Takara, Japan). The qPCR was performed according to the SYBR Premix Ex Taq™ II instructions (Takara, Japan). The reaction was performed on a LightCycler® System (Roche, Germany). Primers for RT-qPCR were synthesized by Shanghai Generay Biotech Co., Ltd. (Supplementary Table S1). Amplifications were performed in triplicate for each sample. The relative abundances of target genes to that of the reference gene (*β*-actin) were calculated according to 2^−ΔΔCt^ method.

### 2.6. Statistical Analysis

The statistical significance of differences among means was assessed with one-way analyses of variance (ANOVA) and Student-Newman-Keuls test. All the data are expressed as mean ± standard deviations (SD).* P *< 0.05 was regarded as statistically significant. The Prism software (Graphpad, San Diego, CA, USA) was used for all statistical analyses.

## 3. Results

### 3.1. MFGM Supplementation during Neonatal Stage Improved the Growth Performance of LBW Mice

**Figure 1:**
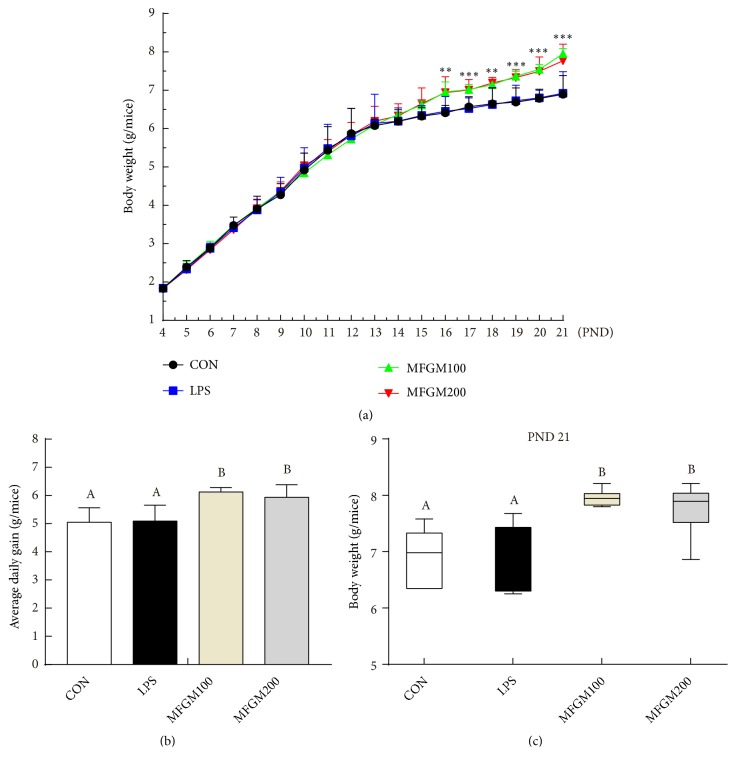
*Effects of MFGM supplementation from postnatal d 4 to d 21 on the body weight gain in LBW mice*. Before LPS being injected, pups were fed with phosphate buffered solution (CON and LPS group), MFGM at 100 mg/kg BW (MFGM100), and 200 mg/kg BW (MFGM200) from PND4 to PND21. (a) Body weight from PND4 to PND21. (b) Average daily gain. (c) The body weight of pups at PND21. Mean values with their standard errors of the mean (SEM) (n = 8 pups/group). Within a row, means without a common letter (A, B, and C) differ (*p* < 0.05).

As shown in Figure 1(a), there was no difference in the body weight of LBW mice among the CON, LPS, MFGM100 and MFGM200 groups at beginning of the experiment (PND4). From PND16 to PND21, the body weight of MFGM100 and MFGM200 groups became significantly higher (*P* < 0.05) than that of the PBS gavaged treatments, due to the higher average daily gain with MFGM treatment (*P* < 0.05) (Figure 1(b)). Finally, at PND21, the body weight of pups from the MFGM100 and MFGM200 groups were higher (*P* < 0.05) (Figure 1(c)) than the pups from the normal diet groups (CON and LPS).

### 3.2. MFGM Presupplementation Prevented the Intestinal Inflammatory Alterations Induced by LPS Challenge in LBW Mice

**Figure 2:**
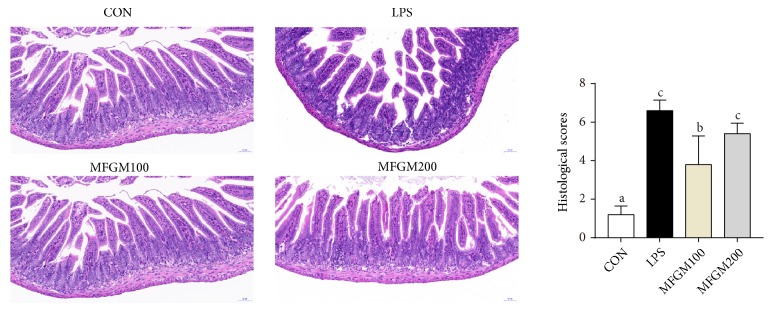
*Effect of MFGM presupplementation from postnatal d 4 to d 21 on ileum damage in LBW mice after LPS challenge*. Mean values with their standard errors of the mean (SEM) (n = 8 pups/group). Within a row, means without a common letter (a, b, and c) differ (*p* < 0.05).

**Figure 3:**
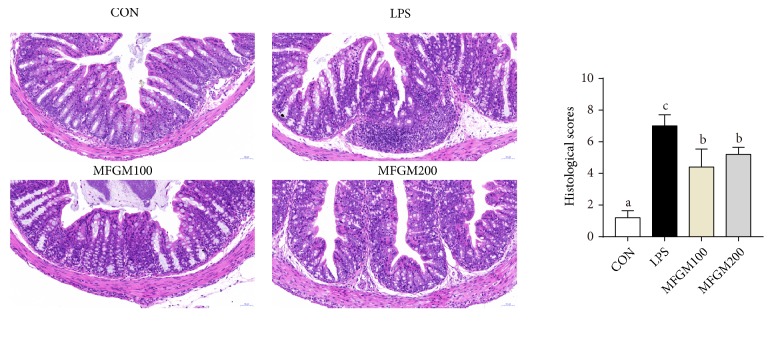
*Effect of MFGM presupplementation from postnatal d 4 to d 21 on colon damage in LBW mice after LPS challenge*. Mean values with their standard errors of the mean (SEM) (n = 8 pups/group). Within a row, means without a common letter (a, b, and c) differ (*p* < 0.05).

As shown in Figures 2 and 3, LPS-challenged mice had increased infiltration of inflammatory cells in the mucosal layer of ileal and colonic tissue compared with the control pup (CON), while these harmful effects of LPS were significantly mitigated (*P* < 0.05) in MFGM100 and MFGM200 groups (Figures 2 and 3), suggesting that MFGM supplementation during the neonatal stage alleviated the LPS-induced intestinal injury in LBW pups. Meanwhile, feeding of the 100 mg/kg BW MFGM showed better effects than MFGM200 in mitigating (*P* < 0.05) the LPS-induced intestinal damage.

To gain an insight into the effects of MFGM on secretional level of inflammatory cytokines in LPS-induced LBW pups, we assessed the concentrations of cytokines including TNF-*α*, IL-6, and IL-1*β* in the plasma. As shown in Table 1, pups challenged with LPS (LPS group) had higher levels of TNF-*α*, IL-6, and IL-1*β* than the CON group (*P* < 0.05), which was largely relieved (*P* < 0.05) by MFGM supplementation, suggesting an effect of MFGM (MFGM100 and MFGM200 group) in inhibiting the secretion of proinflammatory cytokines. Furthermore, the supplementation of 100 mg/kg BW MFGM resulted in significantly lower concentrations of the proinflammatory cytokines (IL-6 and IL-1*β*) (*P* < 0.05) in pups than the MFGM200 group.

**Figure 4:**
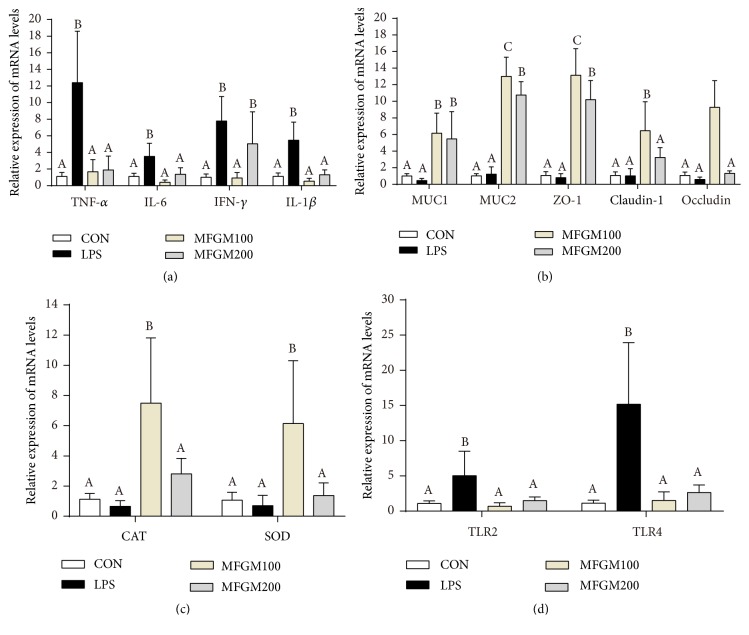
*Effect of MFGM presupplementation from postnatal d 4 to d 21 on the mRNA abundance of genes in the ileum of LBW mice after LPS challenge*. Mean values with their standard errors of the mean (SEM) (n = 8 pups/group). Within a row, means without a common letter (A, B, and C) differ (*p* < 0.05).

**Figure 5:**
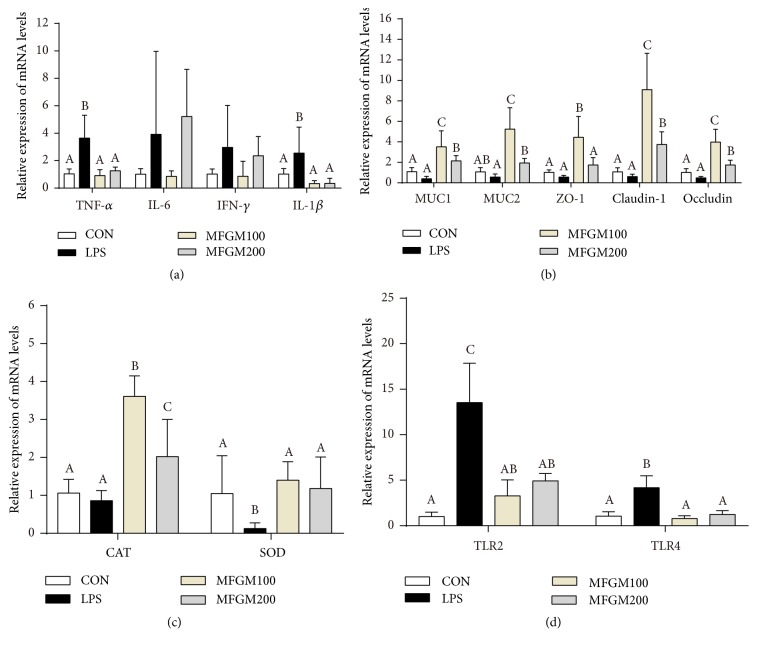
*Effect of MFGM presupplementation from postnatal d 4 to d 21 on the mRNA abundance of genes in the colon of LBW mice after LPS challenge*. Mean values with their standard errors of the mean (SEM) (n = 8 pups/group). Within a row, means without a common letter (A, B, and C) differ (*p* < 0.05).

Expression level of the proinflammatory genes such as TNF-*α*, IL-6, IFN-*γ*, and IL-1*β* was provided in Figures 4 and 5. In the ileum (Figure 4(a)), the pups challenged with LPS had higher expression of TNF-*α*, IL-6, IFN-*γ*, and IL-1*β* (*P* < 0.05), compared to the mice in CON group. However, the MFGM presupplementation (MFGM100 or MFGM200 group) relieved (*P* < 0.05) the LPS-induced intestinal damage by decreasing the gene expression levels of TNF-*α*, IL-6, and IL-1*β*. In the colon (Figure 5(a)), compared to the controls, the genes expressions of TNF-*α* and IL-1*β* were significantly increased (*P* < 0.05) in the LPS group, which were decreased (*P* < 0.05) by MFGM treatment (MFGM100 or MFGM200 group).

### 3.3. MFGM Presupplementation Prevented the Alterations of Plasma Antioxidant Index and Intestinal Antioxidant Gene Expressions Induced by LPS Challenge in LBW Mice

As shown in Table 1, the plasma activity of T-SOD was increased (*P* < 0.05) by MFGM presupplementation compared with the LPS group, and the MFGM100 had higher (*P* < 0.05) activity of T-SOD than MFGM200. LBW pups fed with 100 mg/kg BW MFGM (MFGM100 group) had also higher (*P* < 0.05) mRNA levels of CAT and SOD as compared with the controls in both ileum and colon, whereas there was no difference (*P* > 0.05) between the LPS group and CON group in ileum (Figures 4(c) and 5(c)). However, the expression level of SOD was lower (*P* < 0.05) in colon of the LPS group than those in other groups.

### 3.4. MFGM Presupplementation Regulated the Gene Expressions of Tight Junction and Inflammatory Pathway and Prevented Activation of the Toll-Like Receptors Induced by LPS Challenge in LBW Mice

To investigate whether the MFGM supplementation regulates intestinal tight junction, the gene expressions of ZO-1, claudin-1, and occludin in ileum and colon were measured. As shown in Figures 4 and 5, there were significant interactions (*P* < 0.05) between LPS challenge and intestinal tight junction or MUCs genes expression in ileum and colon. After the LBW pups were injected intraperitoneally with LPS for 4 h, presupplementation of MFGM increased the mRNA expression levels of ZO-1, claudin-1, and occludin (*P* < 0.05) (Figures 4(b) and 5(b)). At the same time, the expression of MUC1 and MUC2 was significantly increased (*P* < 0.05) by MFGM administration (MFGM100 and MFGM200 groups) in both ileum and colon (Figures 4(b) and 5(b)), compared with the CON and LPS group.

Compared with the MFGM200 group, MFGM100 group had higher (*P* < 0.05) mRNA expression levels of ZO-1, claudin-1, and occludin in both ileum and colon. After 4 h of LPS injection (Figures 4(d) and 5(d)), the gene expressions of TLR2 and TLR4 in ileum and colon were higher (*P* < 0.05) in LPS group than those in the CON group, while were significantly decreased (*P* < 0.05) by MFGM presupplementation (MFGM100 and MFGM200 groups).

## 4. Discussion

The gastrointestinal tract (GIT) is of paramount importance in postnatal nutrient digestion and acquisition, where the epithelial barrier of the GIT plays an important function in immune system during neonatal period [26]. However, LBW or IUGR predispose the offspring to malnutrition and endanger the development of intestine and other organs after birth [2, 27, 28]. In current study, our results showed that MFGM supplementation from PND4 to PND21 improved the growth performance of LBW mice, and the MFGM presupplementation mitigated LPS-induced intestinal damage and inflammation at PND21. Our results suggested that this protective effect of MFGM is associated with reduced secretion of proinflammatory cytokines, increased antioxidant enzyme activity, and reduced gene expressions of TLR2 and TLR4. To the best of our knowledge, this is the first to report that MFGM supplementation affects the epithelium integrity and toll-like receptors pathway in LBW neonatal mice.

First, MFGM supplementation from PND4 to PND21 improved the weight gain and growth performance in LBW pups. Studies showed that milk contains various bioactive compounds for infants, playing vital roles in regulating GIT development and protecting against infections during the early life of infants [29]. Milk fat globule membrane is a bioactive molecule better for gut health [13]. MFGM supplementation could increase the villus lengths and decrease crypt depths in the neonatal period of mice and, therefore, usually increase the utilization of nutrients [30, 31]. In this study, our results showed that MFGM supplementation (100 mg/kg BW or 200 mg/kg BW) could improve the growth performance of LBW pups from PND16 to PND21, which is consistent with the previous study [22]. The growth-promoting effects during this time zone could be explained by the increased needs of the neonates for growth but gradually decreased amount of secreted breast milk from PND14 [8, 32]. Consequently, further studies need to be done to elucidate how MFGM supplementation affects the growth performance at different ages.

Second, MFGM presupplementation improved the intestinal structure and barrier function and alleviated the intestinal inflammation after LPS challenge. In link with previous studies [33], intestinal villus was damaged by LPS challenge, suggesting that LPS could cause obstruction of intestinal development and histological damage during early life. As mentioned before, the GIT was important for postnatal nutrient acquisition [26]; damage of the intestinal morphology and structure was associated with disorder of digestion and absorption. In the current study, our results showed that MFGM presupplementation from PND 4 to PND 21 mitigated the intestinal histological damage and decreased the infiltration of intestinal lamina propria in ileum and colon of LBW mice challenged by LPS, which were in accordance with study carried out by Snow et al. [16]. Previous studies have also found that the intestinal epithelium forms the most important barrier among the internal and external environments [34]. The barrier is maintained by the tight junctions, including ZO-1, occludin, and claudin-1 [8, 33, 35]. Meanwhile, mucins (MUCs), the major components of mucous and a kind of glycoproteins, act as various roles in homeostasis [36] and can protect and lubricate the epithelial mucosa [37]. Our results showed that MFGM presupplementation increased the mRNA levels of ZO-1, claudin-1, and occludin in ileum and colon of the LPS-induced LBW mice and improved the expression of MUC1 and MUC2, in accordance with the study carried out by Snow et al. [16]. These results suggested that MFGM supplementation improves the development of villi morphology, as well as maintaining the intestinal integrity and barrier function by improving the expression levels of tight junction and MUCs.

Third, MFGM presupplementation improved the intestinal oxidative stress during LPS challenge. Due to the suddenly increased oxygen concentration at birth, a large number of oxygen free radicals were produced in the intestine [8, 38–40], ultimately leading to oxidative stress in the GIT of the neonates [38, 41]. According to previous studies [8, 10, 28, 38, 42], the impaired GIT of IUGR neonates is prone to produce a large amount of oxygen free radicals, thereby exacerbating their oxidative stress. The intestinal morphology and barrier of neonatal mice were disturbed by LPS, leading to increased intestinal permeability and damaged tight junction, ultimately resulting in oxidative stress [43–45]. Oxidative stress is frequently associated with intestinal epithelial barrier and inflammatory cytokines, such as tight junction and proinflammatory cytokines, respectively [8, 46]. Previous studies showed that oxidative stress could increase the proinflammatory cytokine (TNF-*α*) expression and decreased the tight junction mRNA level in the intestinal mucosa of piglets [16, 46, 47], where oxidative stress could be a vital factor leading to intestinal dysfunction in LBW piglets [8]. To investigate the oxidative stress in LBW pups after LPS challenge, the redox status of plasma and intestine was determined. In the current study, the plasma concentration of SOD was increased by MFGM presupplementation from PND4 to PND21 in LPS-induced LBW mice. Meanwhile, MFGM presupplementation increased the expression levels of SOD and CAT in both ileum and colon after LPS challenge. It was well known that SOD and CAT are antioxidant enzyme systems to maintain cellular integrity and tissue redox homeostasis [48, 49]. And MDA is the most important biomarker of lipid peroxidation products and may impair intestinal integrity and intestinal permeability [8]. Thus, MFGM supplementation could ameliorate intestinal dysfunction in LBW pups by enhancing the antioxidant systems.

Fourth, mammalian toll-like receptors (TLRs) play a vital role in signal transduction during pathogen invasion, inflammatory, and immune response [50, 51]. Previous studies revealed that TLR2 played important role in inflammatory response [50, 51]. Meanwhile, it was well known that intestinal epithelium cells recognize LPS through the membrane protein of TLR4. LPS can cause intestinal inflammatory and promote the expression levels of proinflammatory cytokines via activation of the TLR4/NF-*κ*B signaling pathway [52]. According to our results, the expression levels of TLR4 and TLR2 were increased in the ileum and colon of LPS-challenged LBW mice, while being significantly abolished by MFGM presupplementation. Meanwhile, the genes expression of proinflammatory cytokines was decreased in the MFGM presupplementation group. In summary, our results showed that MFGM presupplementation decreased the expression level of proinflammatory cytokines by modulating TLR signaling pathway in LPS-induced LBW mice, which was consistent with previous studies [16, 33].

In conclusion, using the LPS-induced neonatal LBW mice model, we demonstrated that MFGM supplementation improved the growth performance of the neonatal mice during their early life. MFGM presupplementation could also alleviate the intestinal damage induced by LPS challenge in LBW mice through increasing the mRNA levels of tight junction, intestinal mucosal barrier, and antioxidant enzyme, while reducing the expression of proinflammatory cytokines by inhibiting TLR2 and TLR4 signaling. This study disclosed that MFGM is a functional nutrient with an ability to improve the growth performance of LBW mouse pups, especially during the LPS challenge, and provides a new approach for treatment or prevention of intestinal inflammatory in LBW neonates during their early life.

## Figures and Tables

**Table 1:** Effect of MFGM supplementation from postnatal d 4 to d 21 on plasma proinflammatory cytokines, antioxidant enzyme activity, and oxidant products in LPS-challenged LBW mice^a^.

Items	CON	LPS	MFGM100	MFGM200
TNF-*α* (pg/ml)	19.14±1.80a	37.02±4.84c	27.84±3.16b	24.63±2.51b
IL-6 (pg/ml)	227.03±15.68a	325.00±10.70d	282.04±15.97c	251.99±12.94b
IL-1*β* (pg/ml)	15.56±4.12a	26.01±3.49c	20.14±3.94b	25.69±3.45c
T-SOD (U/ml)	109.69±3.30a	101.45±11.58a	127.18±7.92c	111.70±8.05ab
MDA (nmol/ml)	5.09±0.83	5.38±0.80	5.06±0.83	5.32±0.45

^a^Mean values with their standard errors of the mean (SEM) (n = 8/group). Within a row, means without a common letter (a, b, c, and d) differ (*p* < 0.05).

## Data Availability

The data used to support the findings of this study are available from the corresponding author (jkywjj@hotmail.com) upon request.

## Abbreviations

LBW: Low birth weight
PND: Postnatal day
LPS: Lipopolysaccharides
ANOVA: One-way analysis of variance
IL-1*β*: Interleukin-1 beta
IL-6: Interleukin-6
TNF-*α*: Tumor necrosis factor alpha
IFN-*γ*: Interferon-gamma
SD: Standard deviation
CAT: Catalase
SOD: Superoxide dismutase
TLRs: Toll-like receptors
TLR2: Toll-like receptor 2
TLR4: Toll-like receptor 4
MUC1: Mucin 1
MUC2: Mucin 2.

## Supplementary Materials

Supplementary Materials Supplementary Table S1: the information of primer sequences used for qRT-PCR quantification of related genes.
