# Correction to “Milk Fat Globule Membrane Supplementation Promotes Neonatal Growth and Alleviates Inflammation in Low‐Birth‐Weight Mice Treated With Lipopolysaccharide”

**DOI:** 10.1155/bmri/9872417

**Published:** 2025-11-14

S. Huang, Z. Wu, C. Liu, D. Han, C. Feng, S. Wang, and J. Wang, “Milk Fat Globule Membrane Supplementation Promotes Neonatal Growth and Alleviates Inflammation in Low‐Birth‐Weight Mice Treated With Lipopolysaccharide”, *BioMed Research International* 2019 (2019): 4876078, https://doi.org/10.1155/2019/4876078.

In the article titled “Milk Fat Globule Membrane Supplementation Promotes Neonatal Growth and Alleviates Inflammation in Low‐Birth‐Weight Mice Treated With Lipopolysaccharide”, there was an error in Figure 2 related to an accidental duplication of the image of the ileum tissue of a mouse from the MFGM100 group.

This error was introduced by the author during figure assembly and should be corrected as follows:

**Figure 2:**
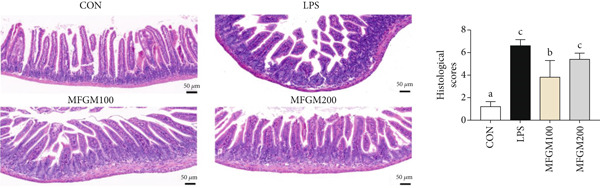
Effect of MFGM presupplementation from Postnatal Day 4 to Day 21 on ileum damage in LBW mice after LPS challenge. Mean values with their standard errors of the mean (SEM) (*n* = 8 pups/group). Within a row, means without a common letter (a, b, and c) differ (*p* < 0.05).

We apoloize for this error.

