# Supplementary material for: Milk Fat Globule Membrane Supplementation Promotes Neonatal Growth and Alleviates Inflammation in Low-Birth-Weight Mice Treated with Lipopolysaccharide
Source: Biomed Res Int. 2019 May 2;2019:4876078. doi: 10.1155/2019/4876078 (PMC6521396; doi:10.1155/2019/4876078)
Supplement: Supplementary Materials — Supplementary Table S1: the information of primer sequences used for qRT-PCR quantification of related genes. [file 4876078.f1.pdf]

Supplementary Table S1. Mice primer sequences used in qRT-PCR

Genes	Forward	Reverse
β-Actin	TGGAATCCTGTGGCATCCATGAAAC	TAAAACGCAGCTCAGTAACAGTCCG
IL-6	ACCACGGCCTTCCCTACTT	CACAACTCTTTTCTCATTTCCAC
TNF-α	TGGGAGTAGACAAGGTACAACCC	CATCTTCTCAAAATTCGAGTGACAA
IFN-γ	GCGGCTGACTGAACTCAGATTGTAG	AGTGCTGTCTGGCCTGCTGTTA
IL-1β	CTCGCAGCAGCACATCAACAAG	GGAAGGTCCACGGGAAAGACAC
MUC2	TGCTGACGAGTGGTTGGTGAATG	GATGAGGTGGCAGACAGGAGACA
MUC1	AATGGCTCCTCGGTGCTACCTA	TGACTTGGCACTGAAGGCTGAG
ZO-1	CGGAACTATGACCATCGCCTAC	CTTCGGGATGTTGTCTGGAGTC
Claudin-1	AGCTGTGCATGGCCTCTTGT	CCAATGTCAATGGCAACACCCT
Occludin	CAGCCTCGGTACAGCAGCAAT	ATAGTGGTCAGGGTCCGTCCTC
TLR2	AAGATGTCGTTCAAGGAGGTGCG	ATCCTCTGAGATTTGACGCTTTG
TLR4	GGTGTGAAATTGAGACAATTGAAAAC	GTTTCCTGTCAGTACCAAGGTTGA
SOD	GAGACCTGGGCAATGTGACT	GTTTACTGCGCAATCCCAAT
CAT	AAATGCTTCAGGGCCGCCTT	GTAGGGACAGTTCACAGGTA
